# Tartarized Sulphate of Quinine

**Published:** 1853-06

**Authors:** 


*Tartarized Sulphate of Quinine.*—Dr. Galamina (Bull. delle Sc. Med., xxi. p. 99) speaks in strong terms of praise of the febrifuge power of sulphate of quinine when combined with equal parts of tartaric acid—a much smaller quantity of the alkaloid so administered sufficing. During an epidemic of ague, it was given in 43 cases, in 31 of which it speedily effected a cure. In 21 of these, half a scruple sufficed, while in 10 others, it required more continued use. In most of the cases, there was hyperæmia of the brain or bronchial membrane, enlarged spleen, or gastro-biliary derangement, requiring the preliminary emylɔyment of bleeding or purgatives. Of the 12 other cases, 5 had relapses; in 3 no effect was produced; and in 4, the above-named irritative symptoms returned.

